# Upper Limbs Muscle Co-contraction Changes Correlated With the Impairment of the Corticospinal Tract in Stroke Survivors: Preliminary Evidence From Electromyography and Motor-Evoked Potential

**DOI:** 10.3389/fnins.2022.886909

**Published:** 2022-06-01

**Authors:** Wenfei Sheng, Shijue Li, Jiangli Zhao, Yujia Wang, Zichong Luo, Wai Leung Ambrose Lo, Minghui Ding, Chuhuai Wang, Le Li

**Affiliations:** ^1^Department of Rehabilitation Medicine, The First Affiliated Hospital of Sun Yat-sen University, Guangzhou, China; ^2^Faculty of Science and Technology, University of Macau, Taipa, Macao SAR, China; ^3^Institute of Medical Research, Northwestern Polytechnical University, Xi’an, China

**Keywords:** stroke, muscle co-contraction, motor-evoked potential, corticospinal tract, correlation analyses

## Abstract

**Objective:**

Increased muscle co-contraction of the agonist and antagonist muscles during voluntary movement is commonly observed in the upper limbs of stroke survivors. Much remain to be understood about the underlying mechanism. The aim of the study is to investigate the correlation between increased muscle co-contraction and the function of the corticospinal tract (CST).

**Methods:**

Nine stroke survivors and nine age-matched healthy individuals were recruited. All the participants were instructed to perform isometric maximal voluntary contraction (MVC) and horizontal task which consist of sponge grasp, horizontal transportation, and sponge release. We recorded electromyography (EMG) activities from four muscle groups during the MVC test and horizontal task in the upper limbs of stroke survivors. The muscle groups consist of extensor digitorum (ED), flexor digitorum (FD), triceps brachii (TRI), and biceps brachii (BIC). The root mean square (RMS) of EMG was applied to assess the muscle activation during horizontal task. We adopted a co-contraction index (CI) to evaluate the degree of muscle co-contraction. CST function was evaluated by the motor-evoked potential (MEP) parameters, including resting motor threshold, amplitude, latency, and central motor conduction time. We employed correlation analysis to probe the association between CI and MEP parameters.

**Results:**

The RMS, CI, and MEP parameters on the affected side showed significant difference compared with the unaffected side of stroke survivors and the healthy group. The result of correlation analysis showed that CI was significantly correlated with MEP parameters in stroke survivors.

**Conclusion:**

There existed increased muscle co-contraction and impairment in CST functionality on the affected side of stroke survivors. The increased muscle co-contraction was correlated with the impairment of the CST. Intervention that could improve the excitability of the CST may contribute to the recovery of muscle discoordination in the upper limbs of stroke survivors.

## Introduction

Stroke is the major disease that leads to mortality and disability worldwide GBD 2016 Stroke Collaborators (2019). The most common impairment of stroke survivors is motor impairment, which affects an individual’s ability to perform everyday activities and participate in social life (Langhorne et al., 2009). Hemiparesis is the most common symptom in stroke survivors (Bourbonnais et al., 1989; Nakayama et al., 1994; Wolfe, 2000; Roger et al., 2012), with abnormal muscle activation patterns being commonly observed (Bourbonnais et al., 1989). In many stroke survivors, motor impairment originates primarily in abnormal muscle coactivation (Dewald et al., 2001). Muscle co-contraction refers to the simultaneous activity of the agonist and antagonist muscles across the same joint (Banks et al., 2017; Souissi et al., 2018). Surface electromyography (EMG) can detect the muscle activities of the agonist and antagonist muscles (Campanini et al., 2020), and it can be used to identify abnormal muscular coordination in stroke survivors (Bourbonnais et al., 1989; Safavynia et al., 2011). The co-contraction between agonist and antagonist muscles can be evaluated quantitatively using the co-contraction index (CI) (Frost et al., 1997; Song and Tong, 2013; Banks et al., 2017; Li et al., 2020). Song and Tong (2013) found that there was an increased co-contraction between agonist and antagonist muscles of elbow during voluntary movement on the affected side compared with the unaffected side in stroke survivors. Increased muscle co-contraction indicates that the muscles could not contract independently (Hu et al., 2013). Hammond et al. (1988) found that the agonist and antagonist muscles of the wrist joint have a higher co-contraction ratio during voluntary isometric contraction on the affected side compared with the healthy control group. Kamper and Rymer (2001) found that stroke survivors had excessive co-contraction of hand muscles compared with the healthy control group. Increased muscle co-contraction leads to impairment in the upper limb motor function in stroke survivors. Previous studies reported that increased muscle co-contraction had a negative effect on voluntary movement (Chae et al., 2002; Chalard et al., 2019). It could bring about increased duration of the movement, muscle discoordination, and decreased range of movement (Arene and Hidler, 2009; Gross et al., 2015; Sarcher et al., 2015). Several studies applied the CI to evaluate muscular coactivation pattern changes during stroke recovery (Hammond et al., 1988; Chae et al., 2002; Hu et al., 2009; Nam et al., 2017; Qian et al., 2017; Rong et al., 2017). Chae et al. (2002) found that the co-contraction between the agonist and antagonist muscles of the wrist showed a negative relationship to motor function of the upper limbs, evaluated by Fugl-Meyer scales and arm motor ability test. Previous studies assessed the structural and functional muscle alternation after stroke by ultrasonography (Kim et al., 2021), muscle biopsy (Dalise et al., 2020), sEMG (Hu et al., 2015), high-density-surface (HD-sEMG) (Tanzarella et al., 2020), and dual-energy X-ray absorptiometry (Choi et al., 2021). There are studies that applied sEMG, kinematic parameters, and clinical scales to evaluate the upper-limb motor function in stroke survivors (Donoso Brown et al., 2014; Pan et al., 2021). But these studies focused only on the changes in the properties of muscles. For better stroke rehabilitation, it is necessary to assess the peripheral muscle changes and alternation in descending motor pathway at the same time (Azzollini et al., 2021).

The corticospinal tract (CST) is the principal neural pathway of the voluntary drive to the upper limb where muscle synergy is modulated (Lemon, 2008; McMorland et al., 2015; Van Wittenberghe and Peterson, 2021). The assessment of CST includes the transcranial magnetic stimulation (TMS) and diffusion tensor imaging (DTI) (Jang, 2013; Potter-Baker et al., 2016). Motor-evoked potential (MEP), elicited by TMS, provides quantitative method for evaluating the functional integrity of the CST (Groppa et al., 2012; Bestmann and Krakauer, 2015; Okamoto et al., 2021). TMS could induce rapidly changing magnetic field that stimulates cortical neurons and generates induced current. The induced current then depolarizes cortical axons and triggers MEP at suprathreshold stimulus intensities. The MEP is transmitted to the peripheral muscle through a descending path such as CST and corticobulbar motor pathways (Groppa et al., 2012). MEP provides insight into the mechanisms of motor output control (Bestmann and Krakauer, 2015) and can be applied to monitor the clinical progression stroke recovery (Cakar et al., 2016). Longer latency, smaller amplitude, and higher thresholds of MEP were observed on the affected side compared with the unaffected side in stroke survivors (Turton et al., 1996; Pennisi et al., 2002). During the recovery from stroke, the MEP of the paresis side changes toward the healthy state (Barker et al., 2012). Byrnes et al. (1999) showed that the MEP had a broad relationship with motor deficit as assessed by the Motor Assessment Scale and British Medical Research Council Scale (Brouwer and Schryburt-Brown, 2006). Bowden et al. (2014) showed that muscle weakness of the upper limb in stroke survivors resulted from the impairment of the descending corticospinal connections. Madhavan et al. (2011) investigated the correlation between the CST integrity and muscle strength by TMS, DTI, and dynamometer. There are few studies combining the assessment of muscle activation with the evaluation of the CST. Although there are many studies using MEP to assess the motor function of the stroke survivors (Turton et al., 1996; Traversa et al., 1998; Hendricks et al., 2003), only a limited number of studies investigated the correlation between MEP and muscle discoordination in stroke survivors.

Hortobágyi and Devita (2006) found that there was increased muscle co-contraction in the agonist and antagonist muscles in older adults. The age-associated change in the muscle co-contraction might result from the cortical component. The increased coactivation between the ankle and knee extensors in the paretic leg of stroke survivors was correlated with alterations in propriospinal pathways (Dyer et al., 2011). Chalard et al. (2020) conducted an EEG study, which found that an increased co-contraction was correlated with cortical movement-related beta oscillation alterations. Increased recurrent Renshaw inhibition is considered to be related to the increased co-contraction of the agonist and antagonist muscles (Katz and Pierrot-Deseilligny, 1982), Another physiological mechanism associated with increased muscle co-contraction of the agonist and antagonist includes the decrease in the Ia reciprocal inhibition, presynaptic inhibition, and Ib inhibition (Morita et al., 2006; Crone et al., 2007; Baude et al., 2019). The decrease in reciprocal inhibition was associated with the impairment of the CST (Crone et al., 2004). Much remains to be understood about the correlation between the impairment of the CST and increased muscle co-contraction of the upper limbs of stroke survivors. The impaired motor function is not only the result of the dysfunction of central motor control system but the result of the alternation in muscle activation (Azzollini et al., 2021). MEP evoked by TMS could reflect the function of the CST. The sEMG data provided the information of the peripheral muscle activity. The correlation between sEMG and MEP from TMS could lead to a better understanding about the mechanism of the abnormal muscle contraction pattern in stroke survivors. Especially, these findings provided insights into the mechanism of increased muscle co-contraction in stroke survivors. Therefore, the study aimed to probe the possible correlation between MEP and CI of the agonist and antagonist muscles during voluntary movement of the upper limbs of stroke survivors. We attempted to investigate whether the abnormal muscle coordination was associated with the impairment of the CST in stroke survivors.

## Materials and Methods

### Participants

Nine stroke survivors and nine age-matched healthy people were recruited after obtaining approval from the Human Subjects Ethics Subcommittee of The First Affiliated Hospital of Sun Yat-sen University. This study is part of the clinical research that was registered on the Chinese Clinical Trial Registry (ChiCTR2000032245). All participants signed written consent prior to participation. The study was performed in accordance with the Declaration of Helsinki. The inclusion criteria of stroke survivors are (1) unilateral stroke; (2) 30–75 years old; (3) the elbow flexors, wrist flexors, and finger flexors scored less than 3 on the Modified Ashworth Scale (MAS); (4) the muscle strength of elbow extensors, wrist extensors, and finger extensors scored more than 2 on the manual muscle testing; (5) no metal implants in brain and cervical spine; (6) has sufficient cognitive ability to follow experimental procedure; and (7) has detectable MEP on abductor pollicis brevis. The exclusion criteria are (1) epilepsy, (2) pregnancy, (3) severe respiratory and circulatory failure, and (4) posterior circulation infarction or posterior circulation hemorrhage.

### Clinical Measures

Fugl-Meyer Assessment Upper Extremity Scale (FMA-UE) and Action Research Arm Test (ARAT) were applied to assess motor function of the upper limb of stroke survivors. The MAS was applied to evaluate the spasticity. All survivors were evaluated by an experienced therapist.

### Electromyography Experiment

**FIGURE 1:**
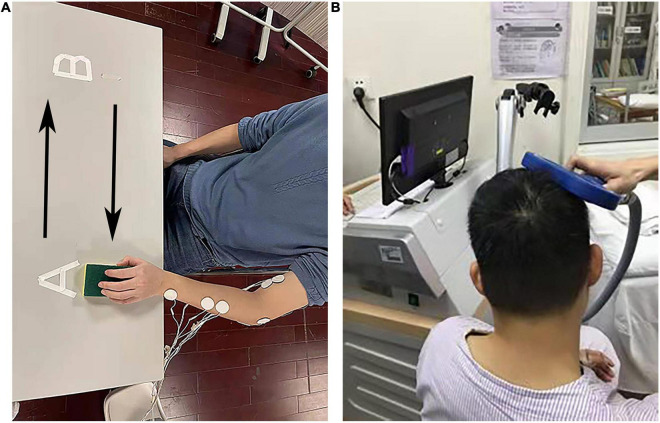
The experimental setup. **(A)** The electromyography (EMG) evaluation during horizontal task. **(B)** The experimental setup for motor-evoked potentials (MEP) evaluation.

After preparing the skin (Nuprep, Weaver and Company, Aurora, CO, United States) and 75% alcohol, four pairs of surface electrodes (Dongguan Quanding Medical Supplies Co., Ltd., Guangdong, China) were placed on the skin surface of four muscle groups in the upper limbs to record EMG signals. The involved muscle groups included flexor digitorum (FD), extensor digitorum (ED), the biceps brachii (BIC), and the triceps brachii (TRI) muscle groups (Figure 1A).

Participants were first directed to perform isometric maximal voluntary contraction (MVC) of the four involved muscles. When a participant conducted the MVC test for the ED and FD, the elbow was kept extended at 130° and the wrist was kept in a neutral position. When a participant conducted the MVC test for the BIC and TRI, the shoulder joint was kept flexed at 45° and the elbow flexed at 90° (Hu et al., 2013; Nam et al., 2017). Each contraction was repeated three times with 2 min intervals. EMG signals were recorded during each MVC test. The EMG data recorded in MVC were used to normalize the RMS recorded in horizontal task.

Then, all the participants were guided to conduct a horizontal task (Figure 1A) for left upper extremity, the participant was instructed to grasp a sponge at point A. Then, the participant held the sponge and transferred it laterally to point B with natural speed. Finally, the participant released the sponge at point B. For right upper extremity, the participant performed the sponge transfer from point B to point A. The distance between point A and point B is 50 cm. The thickness of the sponge is 3 cm. EMG signals were recorded during the whole horizontal task. During the transfer task, the participant kept the testing hand at a height of 2–5 cm from the table top. Each task was repeated three times with 2 min intervals. Figure 1A shows the setup of the horizontal task.

### Motor-Evoked Potential Experiment

Figure 1B shows the setup of the MEP evaluation. A pair of HEX Dual Electrodes (Noraxon U.S.A. Inc., Arizona, United States) were attached to the surface of the abductor pollicis brevis to record the MEP signals. We employed a magnetic stimulator (Yiruide CCY-IA, Wuhan, China) to deliver magnetic stimulation. The stimulation coil was an “8”-shaped coil with a radius of 7 cm. The maximum stimulator output (MSO) of the magnetic stimulator was 2.0 T. The stimulator coil was held over the thumb area of the contralateral motor cortex and C7 cervical spine to elicit MEP signals. The resting motor threshold (rMT), latency and amplitude, and central motor conduction time (CMCT) were measured in bilateral abductor pollicis brevis in the upper limbs of all participants following the published guidelines (Rossini et al., 1994; Kobayashi and Pascual-Leone, 2003; Cakar et al., 2016).

The participants sat on the chair with palms face up on legs. The rMT was defined as the lowest stimulus intensity to evoke liminal MEP, which has an amplitude of at least 50 μV in 5 of 10 continuously trials in resting state. The rMT was expressed as % maximum stimulator output (% MSO) (Rossini and Rossi, 2007). The stimulation intensity on motor cortex was set at 120% of the rMT. The stimulation intensity on C7 cervical spine was set at 80% of the rMT. The CMCT refers to the difference in latency between MEP elicited by the cortical stimulation and MEP produced by spinal stimulation. The MEP signals were captured and amplified with a resolution of 200 μV, signals were then filtered with a bandpass of 10 Hz–2 kHz, and a noise eliminator of 50 Hz (Groppa et al., 2012).

### Data Processing and Analysis

**FIGURE 2:**
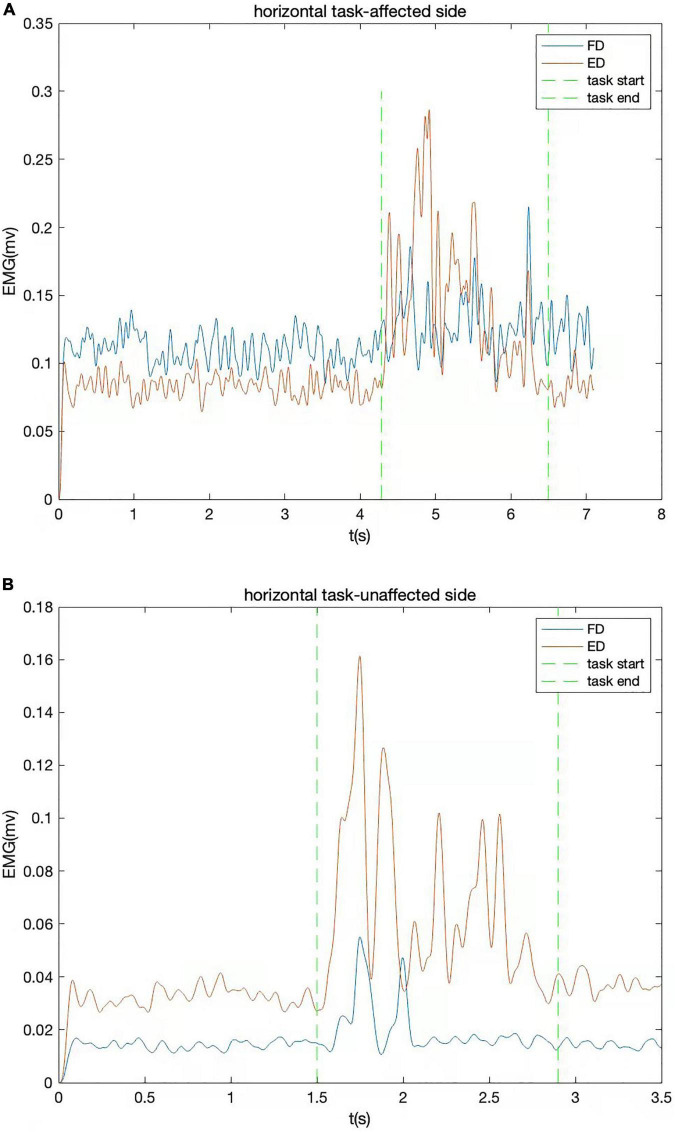
The EMG signals of a typical trial from one stroke survivor captured during horizontal task. The EMG signal of flexor digitorum (FD) and extensor digitorum (ED) are shown together. **(A)** Affected side; **(B)** unaffected side.

All raw EMG data were amplified 1,000 times (amplifier: INA 333, Texas Instruments Inc., Dallas, TX, United States) and sampled with 1,000 Hz for digitization with a data acquisition card (DAQ, 6218 NI DAQ card; National Instruments Corp., Austin, TX, United States). Then, the digitized EMG signals were transferred to the computer for storage. The signals were processed by removing bias, bandpass filtering (bandwidth range from 40 to 490 Hz), full-wave rectification. Lowpass filtering (30 Hz cutoff frequency with fourth-order zero-phrase Butterworth filter) was applied to have the linear EMG envelopes. A typical trial of linear envelopes of the EMG signals captured during horizontal task is shown in Figure 2. EMG data recorded during horizontal tasks were normalized to the maximum value emerging during the MVC test of each muscle. The maximum value of EMG activity was often found in the MVC test, but when higher amplitude of EMG activation emerged in horizontal task, this value would be substituted to be used for normalization. The muscle firing moment was identified as the abrupt moment of EMG signal activation that was greater than the threshold value (Figure 2). The threshold value is calculated by the mean of the EMG baseline plus two times the standard deviation, lasting for 20 ms.

The root mean square (RMS) was calculated to assess the EMG activation level of each muscle (Chae et al., 2002). The RMS value was computed from the EMG data section from the firing moment to the point when the task was finished. The RMS value was calculated by the following expression:


$${\text{V}}_{\text{RMS}}={\left[\frac{1}{T}\int v^{2}\mathrm{dt}\right]}^{1/2}$$

Where T is the length of the signal, and v is the voltage of the EMG signal (Chae et al., 2002). The RMS value could be applied to evaluate the neuromuscular system and the workload on muscles (Moritani, 1993; Shimomura et al., 1999). CI was calculated to assess the degree of muscle co-contraction (Hu et al., 2012, 2013; Nam et al., 2017). The CI value was calculated by the formula previously presented in Frost’s study (Frost et al., 1997):


$$CI=\frac{1}{T}\int_{0}^{T}A_{ij}\left(t\right)dt$$

Where A_*ij*_ (t) is the overlapping activity of the muscles of *i* and *j* in the EMG envelopes, and T is the duration of the task. The CI value varied from 0 to 1. When the activities of two muscles were fully overlapping and the level of EMG activity kept at 1 during the task, the CI value reached 1. When the activities of two muscles did not overlap at all during the task, the CI value was 0.

### Statistical Analysis

The Shapiro–Wilk test was employed to verify the data normality. The difference of EMG parameters and MEP parameters among the groups (affected side, unaffected side, and healthy group) was analyzed by one-way analysis of variance (ANOVA). Bonferroni test was applied to perform *post hoc* pairwise comparison. When the data did not conform to a normal distribution, we transformed the data by natural logarithm or taking cosine until the data were normally distributed. Correlation analysis was applied to analyze the correlation among clinical scale outcomes, CI, and MEP parameters. For data with a normal distribution, Pearson’s correlation analysis was adopted. For data that do not conform to a normal distribution, Spearman’s correlation analysis was employed. The significant level was set at *p* < 0.05. The data analysis was performed with the software SPSS Statistics 26 (IBM Inc., Seattle, WA, United States).

## Results

The characteristics of stroke survivors included in the study are summarized in Table 1.

**TABLE 1:** Clinical characteristics of stroke survivors.

Subject	Age	Gender	Paralyzed side	Stroke type	Month since stroke	FMA-UE	ARAT	MAS (wrist)
1	57	Male	Left	Ischemia	4	53	38	1
2	68	Male	Left	Hemorrhage	2	66	57	0
3	38	Male	left	Hemorrhage	9	32	14	1+
4	31	Male	Left	Hemorrhage	2	61	35	0
5	69	Male	Right	Ischemia	2	54	38	1+
6	72	Female	Left	Ischemia	2	49	33	1
7	66	Male	Left	Ischemia	2	63	54	0
8	31	Male	Left	Hemorrhage	2	62	38	0
9	71	Male	Left	Ischemia	10	45	21	1+

*FMA-UE, Fugl-Meyer Assessment Upper Extremity Scale; ARAT, Action Research Arm Test; MAS, Modified Ashworth Scale.*

### Electromyography Parameters

**FIGURE 3:**
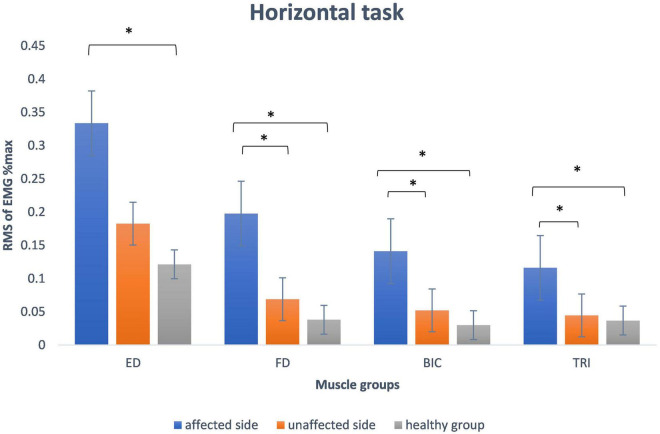
The mean and standard deviation of root mean square of EMG data of all participants during horizontal task involved in the study. The significant difference between the groups is indicated by (*p < 0.05 with one-way ANOVA). ED, extensor digitorum; FD, flexor digitorum; BIC, biceps brachii; TRI, the triceps brachii.

Figure 3 presents the mean and standard deviation of normalized RMS values of the four muscles (FD, ED, BIC, and TRI) during horizontal task. The RMS values of the muscles (FD, BIC, and TRI) on the affected side were significantly higher than the unaffected side in stroke survivors (*p* < 0.05) and the healthy group (*p* < 0.05). The RMS values of the four muscles of the unaffected side of stroke survivors were higher than healthy people but without a significant difference (*p* > 0.05).

Table 2 presents the CI of ED and FD. The CI between two muscles of ED and FD during horizontal task on the affected side was significantly higher than those from the unaffected side (*p* < 0.05) in stroke survivors and the healthy group (*p* < 0.05). The CI of the two muscles of ED and FD in the unaffected side of survivors were higher than those in healthy group but do not reach significant level (*p* > 0.05).

**TABLE 2:** The mean (M) and standard deviation (SD) of CI and MEP parameters of stroke survivors and the healthy group.

	Affected side (M ± SD)	Unaffected side (M ± SD)	Healthy group (M ± SD)
CI (horizontal task)	0.69 ± 0.13^ab^	0.46 ± 0.18	0.44 ± 0.11
MEP latency (ms)	27.59 ± 5.14^ab^	22.15 ± 1.67	22.19 ± 2.23
Amplitude (μV)	196.02 ± 163.68^ab^	565.48 ± 334.98	366.85 ± 119.71
rMT (% MSO)	70.11 ± 19.64^ab^	39.78 ± 6.67	47.33 ± 8.31
CMCT (ms)	12.46 ± 6.12^ab^	7.32 ± 2.20	7.81 ± 1.33

*MEP, motor-evoked potential; rMT, resting motor threshold; CMCT, central motor conduction time.*

*^a^p < 0.05 compared to the unaffected side.*

*^b^p < 0.05 compared to the dominant side of healthy group.*

### Motor-Evoked Potential

Table 2 shows the mean and standard deviation of MEP parameters among the three groups. The MEP latency of the affected side of stroke survivors was significantly longer than the unaffected sides (*p* < 0.05) and the dominant side of healthy people (*p* < 0.05). The MEP amplitude of the affected side of stroke survivors was significantly lower than the unaffected sides (*p* < 0.05) and the dominant side of healthy people (*p* < 0.05). The MEP threshold of the affected side of stroke survivors was significantly higher than the unaffected sides (*p* < 0.05) and the dominant side of healthy people (*p* < 0.05). The MEP CMCT of the affected side of the survivors were significantly longer than the unaffected sides (*p* < 0.05) and the dominant side of healthy people (*p* < 0.05). There was no significant difference in all four MEP parameters between the unaffected sides with the dominant side of healthy people (*p* > 0.05).

### Motor-Evoked Potential and Clinical Scales

**FIGURE 4:**
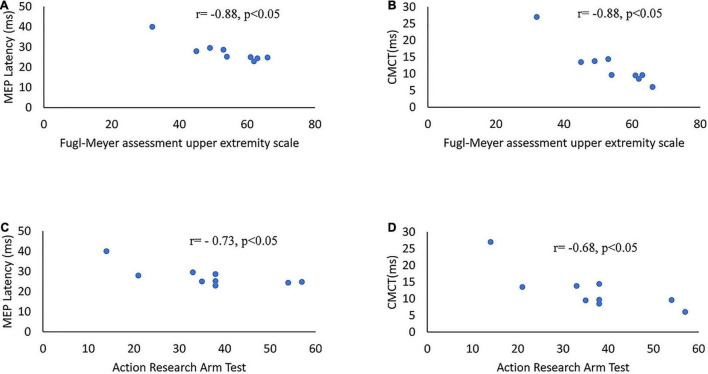
Scatter diagrams of clinical scales and MEP parameters on the affected side of stroke survivors. **(A)** MEP latency versus Fugl-Meyer assessment upper extremity scales. **(B)** CMCT of MEP versus Fugl-Meyer assessment upper extremity scales. **(C)** MEP latency versus ARAT. **(D)** CMCT of MEP versus ARAT. ARAT, Action Research Arm Test; CMCT, central motor conduction time.

Table 3 presents the correlation between MEP parameters and the outcomes of clinical scales. The latency and CMCT of MEP on the affected side were negatively correlated with the upper-limb motor function that was assessed by both clinical scales of Fugl-Meyer scale and ARAT. The amplitude and rMT of MEP showed no significant correlation with the outcome of both clinical scales of Fugl-Meyer scale and ARAT (Figure 4 and Table 3).

**TABLE 3:** Correlation analyses between the motor function measures of affected limb, CI, and the MEP parameters of affected hemisphere.

	FMA-UE	ARAT
CI	−0.318 (*P* = 0.405)	−0.433 (*P* = 0.245)
MEP latency (ms)	−0.883 (*P* = 0.02)	−0.729 (*P* = 0.026)
Amplitude (μV)	0.117 (*P* = 0.765)	0.153 (*P* = 0.695)
rMT (% MSO)	−0.406 (*P* = 0.279)	−0.504 (*P* = 0.166)
CMCT (ms)	−0.883 (*P* = 0.02)	−0.678 (*P* = 0.045)

*FMA-UE, Fugl-Meyer Assessment Upper Extremity Scale; ARAT, Action Research Arm Test; CI, co-contraction index; MEP, motor-evoked potential; rMT, resting motor threshold; CMCT, central motor conduction time.*

### Root Mean Square and Motor-Evoked Potential

The latency of MEP was positively correlated with the RMS value of EMG activity of FD (*r* = 0.52, *p* = 0.033), BIC (*r* = 0.667, *p* = 0.003), and TRI (*r* = 0.578, *p* = 0.015) muscles during horizontal task in stroke survivors. The RMS value of ED during horizontal task was not significantly correlated with the latency of MEP (*r* = 0.245, *p* = 0.343).

### Co-contraction Index and Motor-Evoked Potential

**FIGURE 5:**
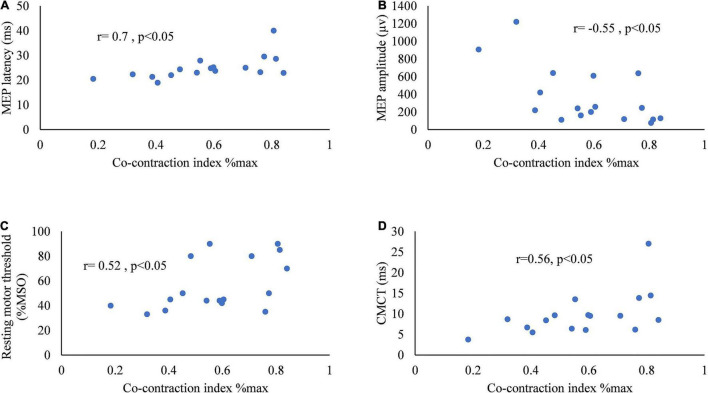
Scatter diagrams of the co-contraction index of flexor digitorum and extensor digitorum during horizontal task versus **(A)** MEP latency, **(B)** amplitude of MEP, **(C)** resting motor threshold of MEP, and **(D)** central motor conduction time of MEP.

Figure 5 presents the correlation between CI and MEP parameters. The CI of the two muscles of ED and FD during horizontal task was positively correlated with latency (*r* = 0.7, *p* < 0.05), rMT (*r* = 0.52, *p* < 0.05), and CMCT (*r* = 0.56, *p* < 0.05) of the MEP in stroke survivors. The CI of the muscles of ED and FD increased along with the increase in latency, rMT, and CMCT of the MEP. The CI of the two muscles of ED and FD during horizontal task was negatively correlated with the amplitude of the MEP (*r* = –0.55, *p* < 0.05) in stroke survivors. The CI of the muscles of ED and FD increased as the amplitude of the MEP decreased.

## Discussion

The main finding of this study indicated that muscle activation alternation of the upper limb was correlated with the changes in the function of CST in stroke survivors. The result provided insights into the origin of the increased muscle co-contraction in the upper limb of stroke survivors. The latency and CMCT of MEP on the affected side in stroke survivors were negatively correlated with the Fugl-Meyer upper extremity scale and ARAT. The CI of the muscle pair of ED and FD during horizontal task was significantly correlated with MEP parameters in stroke survivors. The CI increased, along with an increase in latency, rMT, and CMCT of the MEP. The CI increased along with a reduction in the amplitude of the MEP in the upper limbs of stroke survivors.

### The Differences of Root Mean Square Value and Co-contraction Index Among Groups

The RMS values recorded from the four muscles of the affected side were higher than the unaffected side in the stroke group and also the healthy group during horizontal task. Canning et al. (2000) found that there was a higher biceps muscle activation during a tracking task in the paretic upper limb of stroke survivors compared to the healthy group. Lee et al. (2015) reported an increase in muscle activations on the affected side during a drinking task compared with the unaffected side. Wagner et al. (2007) found that there was higher level of muscle activity during a reaching task on the affected side of the upper limb of stroke survivors than healthy individuals. The results of our study are consistent with previous studies that showed increased muscle activation on the affected side during task execution in stroke survivors. Hu et al. (2013) found a reduction in the upper limb muscles EMG activity that corresponded with upper limbs functional improvement after a 20 sessions training program. The reduction in EMG activities post training was proposed to be the result of a reduction in spasticity that contributed to a lower level of muscle activities (Hu et al., 2007, 2013). The other possible reason was that training sessions contributed to an increase in muscle force production which enabled survivors to perform tasks with less muscle effort (Hu et al., 2007). These findings supported that muscle activation level during task could assist the assessment of clinical progression in motor function during the recovery from stroke. The CI of the muscle pairs of FD and ED on the affected side was higher than the unaffected side in the stroke group and the healthy group during horizontal task. The result was in agreement with previous studies which recorded EMG signals during isometric movement (Hammond et al., 1988; Kamper and Rymer, 2001; Chae et al., 2002). Silva et al. (2014) found that the co-contraction ratio of the proximal upper extremity muscles increased during reaching movement on the affected side of stroke survivors. In contrast to the previous studies, we investigated the abnormal muscular coordination pattern during horizontal task which was more similar to the movement pattern of some daily activities. Song and Tong (2013) reported an increase in co-contraction between the BIC and TRI of the affected side during a tracking task. The excessive co-contraction between the agonist and antagonist reflects the reduction in the control of muscle activity of the affected side in stroke survivors (Song and Tong, 2013). Hu et al. (2013) showed that the CI of ED and FD during horizontal task decreased, along with an improvement in motor function during the recovery from stroke. The result indicated the CI of ED and FD during task could assist in the monitoring of clinical progression of the upper limbs during the recovery from stroke.

### The Difference in Motor-Evoked Potential Parameters Among Groups

The latency, rMT, and CMCT of MEP of the affected side of the stroke group were higher than the unaffected side and the healthy group. The amplitude of MEP was smaller on the affected side. These results were consistent with previously published studies (Turton et al., 1996; Traversa et al., 1997, 1998; Byrnes et al., 1999; Pennisi et al., 2002; Brouwer and Schryburt-Brown, 2006; Barker et al., 2012). MEPs are efficacious in evaluating the CST functionality (Di Lazzaro et al., 1999). The difference of MEPs between groups indicated impairment in the CST functionality. Cakar et al. (2016) showed that latency was negatively correlated with functional outcomes improvement. Cakar et al.’s (2016) results were consistent with our study, where the latency of the MEP was negatively correlated with motor function of the upper limb of stroke survivors. Besides, Cakar et al. (2016) reported that the rMT and amplitude of MEP were correlated with the clinical outcomes of Brunnstrom motor stage, Motricity index, finger tapping test, and motor activity log. However, this study did not observe significant correlation between rMT and Fugl-Meyer scale and ARAT, or between MEP amplitude and Fugl-Meyer scale and ARAT. The amplitude of MEP could be influenced by various factors such as the intensity of stimulation, the condition of intent muscle, and the condition of EEG phase and power fluctuations (Rossini et al., 2015). The rMT of MEP could also be impacted by drugs, age, the intent muscle, and sleep-wake cycles (Groppa et al., 2012). These might be the potential explanations for the different results observed between Cakar et al.’s study and this study. The amplitude and rMT have relatively higher intraindividual variations.

Okamoto et al. (2021) found that the CMCT had a negative relationship with the ARAT score. Cakar et al. (2016) showed that the CMCT of MEP was correlated with the clinical outcomes of Barthel Index, Brunnstrom motor stage, finger tapping test, and motor activity log. Higher motor thresholds and smaller amplitude might result from a loss of corticomotoneurons in the corticospinal pathway and reduced excitability of the motor cortex (Byrnes et al., 1999; Brouwer and Schryburt-Brown, 2006). Thus, MEP latency and CMCT may be appropriate indicators to evaluate the motor function of the upper limb.

### The Correlation Between Electromyography and Motor-Evoked Potential Parameters

Wagner et al. (2007) suggested that the increased level of muscle activities on the affected side of stroke survivors might have originated from extra muscle units recruitment owing to the loss of the functional motor units. In this study, the RMS values of the FD, BIC, and TRI during horizontal task were correlated with the latency of MEP. The results indicated that the increased muscle activity during voluntary movement was correlated with the impairment of the CST. The RMS value of ED showed no significant correlation with the latency of MEP. The possible reason was that the ED muscle took part in a small part of the horizontal task.

The latency of MEP is thought to reflect the conduction time for the neural impulses from the cortex to peripheral muscles (Groppa et al., 2012; Bestmann and Krakauer, 2015). In this study, the CI of FD and ED during horizontal task were positively correlated with the latency of MEP. Shorter latency of MEP corresponded to smaller CI value, which reflected better muscle coordination. The MEP signals involved in the study were recorded from the muscle of the abductor pollicis brevis. Thus, the latency of MEP recorded at FD and ED would be also prolonged due to the anatomical location. The result suggested that the level of the muscular coordination in the upper limb of stroke survivors was correlated with the impairment of cortical transmission.

The result indicated muscle co-contraction of the upper limb of stroke survivors was correlated with the loss of corticospinal projections. The amplitude of MEP is considered to reflect the integrity of the CST and the excitability of the motor cortex (Groppa et al., 2012; Bestmann and Krakauer, 2015). The amplitude of MEP could reflect the transsynaptic excitation of corticospinal cells (Ziemann et al., 2015). Cakar et al. (2016) found that MEP amplitude was positively correlated with the outcomes of motor performance and dexterity of the upper limb. In our study, the CI of FD and ED during horizontal task was negatively correlated with the amplitude of MEP. The lower amplitude of MEP corresponded to an increase in CI. The reduction in MEP amplitude reflected the loss of corticospinal projections (Hömberg et al., 1991; Pennisi et al., 2002).

The rMT of MEP is considered to reflect the integrated excitability of the corticomotor projection (Groppa et al., 2012). Lower rMT was correlated with better motor performance (Cakar et al., 2016). For the CI of a muscle pair of a joint, lower CI value suggested a separation of the co-contraction phase which means the muscle pair could contract more independently (Hu et al., 2013). The CI of FD and ED during horizontal task was correlated with the rMT of MEP in our study. The increase in rMT could be caused by the decrease in the CST excitability which might have originated from loss of the corticospinal projection (Caramia et al., 1991; Pennisi et al., 2002). The result indicated the reduction in the CST excitability might be the possible mechanism to increase muscle co-contraction in the upper limbs of stroke survivors. Recently, Hammerbeck et al. (2021) found that the degree of the CST connectivity is the principal determinant of proximal dexterity strength and muscle synergy in upper limbs of patients with subacute stroke. The result of this study indicated that muscle co-contraction of FD and ED was correlated with the excitability of the CST.

Moreover, the result of this study provided evidence to support that CMCT positively correlated with muscle coherence. Longer CMCT corresponded to higher CI. The CMCT is the most related electrophysiological maker to evaluate the integrity of the CST (Groppa et al., 2012). The increase in CMCT might have originated from the loss of the fast corticospinal fibers and damage to the axonal (Misra and Kalita, 1995; Pennisi et al., 2002; Groppa et al., 2012). The result suggested that the increased muscle co-contraction of the upper limb was correlated with the loss of the corticospinal projection.

The correlation between CI and MEP parameters indicated that increased muscle co-contraction was correlated with the impairment of the CST in the upper limb of stroke survivors. Chae et al. (2002) suggested the underlying mechanisms for the increase in muscle co-contraction included an increase in alpha motoneuron excitability and increased activity in brainstem pathways after damage to the CST and cortical reorganization (Ohn et al., 2013). Chalard et al. (2020) found the increased muscle co-contraction was associated with a reduction in movement-related beta desynchronization. Other various physiological mechanisms such as reduction in Ia reciprocal inhibition, decrease in presynaptic inhibition, and reduction in Ib inhibition were considered to be correlated with an increase in muscle co-contraction in stroke survivors (Baude et al., 2019). The decrease in reciprocal inhibition was correlated with the impairment of the CST (Crone et al., 2004). We propose that the increased muscle co-contraction might have a cortical origin that was correlated with the impairment of the CST. Interventions that facilitate the recovery of the CST function might accelerate the recovery of the muscle coordination in the upper limb after stroke. An example of this type of intervention is neuromuscular electrical stimulation that could enhance the excitability of the CST and facilitate function recovery in stroke survivors (Ridding et al., 2001; Mang et al., 2010; Rong et al., 2015). Studies showed that neuromuscular electrical stimulation could improve the muscular coordination and clinical outcomes (Hu et al., 2009, 2012, 2013; Rong et al., 2015, 2017; Nam et al., 2017). The result of this study provided further evidence for the application of the neuromuscular electrical stimulation in the rehabilitation of muscle dyscoordination in the upper limb of stroke survivors. The correlation between CI and MEP parameters indicated that MEP could be applied to assess the muscle co-contraction in the upper limbs of stroke survivors.

### Limitation

This is a preliminary study and the sample size is limited, which might contain type II error. Part of the survivors involved in the study were chronic stroke survivors. Further studies are required to verify the result in early stroke survivors. Stroke survivors at different stages could have different muscle contraction patterns. The stroke survivors at later stages such as more than 6 months could accompany with compensatory movement. The compensatory movement was proved to have originated from cortical compensatory neuroplasticity. The cortical compensatory neuroplasticity might influence the conclusion. The survivors involved in the study had mild to moderate impairment of the upper-limb function. Further studies are required to verify the results in stroke survivors with severe impairment. The stroke survivors with posterior circulation infarction or hemorrhage were excluded from the study. Future studies are required to verify the result in this group of stroke survivors.

## Conclusion

We demonstrated a statistically significant correlation between muscle co-contraction and the CST function in stroke survivors. The correlation between CI and MEP parameters indicated the CST and peripheral muscle coordination were closely correlated in stroke survivors. Interventions that could increase the excitability of the CST might facilitate the recovery of muscle coordination in the upper limb after stroke.

## Data Availability Statement

The datasets analyzed during the current study are available from the corresponding authors upon reasonable request.

## Ethics Statement

The studies involving human participants were reviewed and approved by the Human Subjects Ethics Subcommittee of The First Affiliated Hospital of Sun Yat-sen University. The patients/participants provided their written informed consent to participate in this study.

## Author Contributions

LL and CW conceived and designed the study. WS, SL, and JZ performed the experiments. WS, YW, and ZL analyzed the data. WS, SL, and WL wrote the manuscript. MD, CW, and LL contributed to experiments. WL, MD, CW, and LL reviewed and edited the manuscript. All authors read and approved the manuscript.

## Conflict of Interest

The authors declare that the research was conducted in the absence of any commercial or financial relationships that could be construed as a potential conflict of interest.

## Publisher’s Note

All claims expressed in this article are solely those of the authors and do not necessarily represent those of their affiliated organizations, or those of the publisher, the editors and the reviewers. Any product that may be evaluated in this article, or claim that may be made by its manufacturer, is not guaranteed or endorsed by the publisher.
